# Prognosis of patients with laryngeal contact granuloma: Development and validation of RCGSG score

**DOI:** 10.1002/lio2.969

**Published:** 2022-11-02

**Authors:** Yufei Pan, Jinrang Li, Xiaoyu Wang, Jinhong Zhang, Chun Zhang, Zhi Liu

**Affiliations:** ^1^ Department of Otolaryngology – Head and Neck Surgery The Sixth Medical Center of PLA General Hospital of Beijing Beijing China

**Keywords:** laryngeal contact granuloma, local glucocorticoid injection, model, prognosis

## Abstract

**Objective:**

This study aimed to develop an objective and simple score for predicting the prognosis of patients with laryngeal contact granuloma (LCG) treated with local glucocorticoid injection combined with oral proton pump inhibitor (GI + PPI).

**Methods:**

Cox regression analysis was used to analyze the effect of baseline variables on the prognosis of 507 patients with LCG treated with GI + PPI. An easy‐to‐apply RCGSG (**R**eflus, **C**ough, **G**ender, and **S**urgery in **G**I + PPI therapy) score was developed based on the independent risk factors selected by univariate and multivariate Cox regression analyses. The score was internally validated by receiver‐operating characteristic curve, calibration curve, and decision curve analysis.

**Results:**

After univariate and multivariate analyses, male gender (hazard ratio [HR] 0.546, *p* < .001), laryngopharyngeal reflux (HR 0.702, *p* = .001), chronic cough (HR 0.709, *p* = .001), and history of surgical resection (HR 0.433, *p* < .001) were found to be the independent risk factors affecting the prognosis of LCG. According to the score, the median cure time was 3 months (95% confidence interval [CI] 2.81–3.19) in the low‐risk group, 4 months (95% CI 3.74–4.26) in the moderate‐risk group, and 5 months (95% CI 4.76–5.24) in the high‐risk group. The bootstrap method was used to plot calibration curves for internal validation.

**Conclusion:**

The RCGSG score, developed based on laryngopharyngeal reflux, chronic cough, gender, and surgical resection history, has been internally verified to be a good predictor of the prognosis of patients with LCG receiving GI + PPI treatment.

**Level of evidence:**

Level 4.

## Graphical abstract

The RCGSG score, developed based on laryngopharyngeal reflux, chronic cough, gender, and surgical resection history, has been internally verified to be a good predictor of the prognosis of patients with laryngeal contact granuloma.

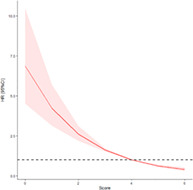

## 1 INTRODUCTION

Laryngeal contact granuloma (LCG) is a relatively rare inflammatory hyperplastic lesion; its treatment has been an issue for otolaryngologists since it was first reported by Jackson et al. in 1928.^1^ Many treatment options are available, including anti‐reflux medications, voice training, glucocorticoid injection (GI), botulinum toxin injection, surgery, and so on. However, some patients could not be treated, irrespective of the option chosen; some scholars refer to it as “refractory laryngeal granuloma.”^2^, ^3^

Wang et al., in 2013, first proposed GI as a treatment option for patients who failed conservative treatment.^4^ Subsequent studies continued to demonstrate the efficiency of GI. In 2016, Tian et al. modified the option to include local GI combined with oral proton pump inhibitor (GI + PPI), further improving the cure rate.^5^ However, treating some patients was still difficult. Also, the long‐term repeated injections caused a lot of pain to patients, eventually leading to treatment withdrawal in some cases. Both patients and otolaryngologists urgently need a predictive score that can help predict the outcome before choosing GI + PPI for treatment, so as to avoid treatment failure.

Therefore, in the present study, we developed and validated a simple and easy‐to‐apply score to predict the outcome of patients with LCG treated with GI + PPI.

## 2 MATERIAL AND METHODS

### 2.1 Patients and inclusion criteria

The clinical data of 507 patients with LCG, who were treated with GI + PPI in the Sixth Medical Center of PLA General Hospital from April 2014 to February 2022, were reviewed in this study. The inclusion criteria were as follows: (1) LCG diagnosed by three otolaryngologists professionals after examining the laryngoscopy images and (2) detailed and complete patient data available. The exclusion criteria were as follows: (1) patients with interrupted treatment and (4) the follow‐up being more than 6 months after cure. The ethics committee of the Sixth Medical Center of PLA General Hospital approved this study.

### 2.2 Treatment and evaluation method

**FIGURE 1:**
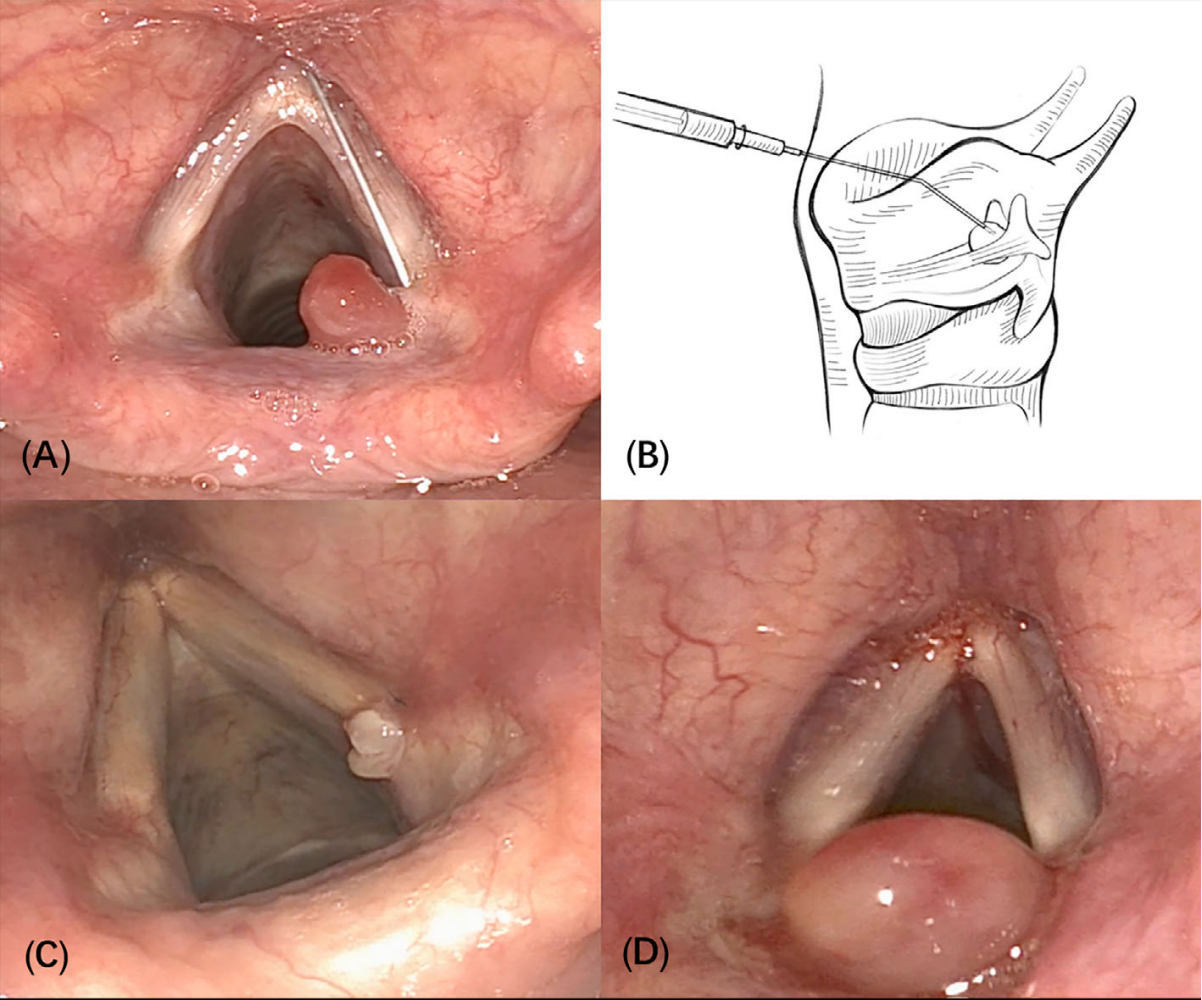
(A) Local glucocorticoid injection (laryngoscopy image). (B) Local glucocorticoid injection (diagram). (C) The Farwell granuloma endoscopic grading system of 1–2 formed the relatively small group. (D) The lesions with the Farwell granuloma endoscopic grading system of 3–4 formed the larger group

Local GI combined with oral proton pump inhibitor (GI + PPI): After local anesthesia, a needle was inserted into the laryngeal cavity via percutaneous puncture along the superior notch of the thyroid cartilage under the surveillance of a transnasal fiberoptic laryngoscope, and triamcinolone acetonide 0.3–1.0 mg was injected into the lesion and the basal cartilage membrane, once a month (Figure 1A,B). Simultaneous oral doses of omeprazole tablets (20 mg) or standard doses of other PPIs were given once each in the morning 30 min before meals.^5^ The patients were followed up once a month for 6 months from the first treatment. Outcome event was defined as the time from the start of treatment to cure (disappearance of the lesion on review) or to the end of the 6‐month follow‐up period. Patients not cured after 6 months of treatment were defined as “treatment failure.” During treatment, we advised all patients to avoid severe coughs and unhealthy lifestyle habits that could lead to laryngopharyngeal reflux. Cumulative cure (CC) time was defined as the time to cure within the 6‐month follow‐up period, or the total time to cure if the disease remained uncured beyond the follow‐up period.

The size of lesions was divided into two groups: the lesions with the Farwell granuloma endoscopic grading system of 1–2 formed the relatively small group, and the lesions with the Farwell granuloma endoscopic grading system of 3–4 formed the larger group. (Figure 1C,D). Grading system has been proposed by Farwell et al6 for contact granuloma on the basis of its endoscopic appearance.^6^ Laryngopharyngeal reflux was evaluated using the laryngopharyngeal reflux symptom index (RSI) and laryngopharyngeal reflux finding score (RFS). RSI >13 and RFS >7 indicated laryngopharyngeal reflux. Chronic cough was defined as a cough lasting more than 8 weeks, with no imaging abnormalities and with cough as the main or only symptom.^7^

### 2.3 Study design and statistics

The following items were included to develop a reliable and clinically generalizable predictive score, referring to previous studies: gender, lesion size, laryngopharyngeal reflux, chronic cough, history of surgical resection, and history of intubation factors. The baseline characteristics were summarized using descriptive statistical methods. Univariate and multivariate analyses were conducted with Cox regression models. Variables with a *p* value <.05 in the univariate analysis were considered for multivariate analysis. The baseline variables with a *p* value <.05 were considered independent risk factors for prognosis. The RCGSG scores were assigned according to the independent risk factors and regression coefficients in the multivariate analysis. The scores were divided into three risk groups based on the cutoff derived from the restricted cubic spline and clinical requirements. The survival curves for different risk groups were plotted by the Kaplan–Meier method.

Since this study was a single‐center study and lacked external data, we only performed internal model validation. The area under the curve (AUC) of the receiver‐operating characteristic (ROC) curve was used to evaluate the score discrimination. A calibration curve was drawn to assess the model calibration. The decision curve analysis was used to measure the potential net benefit of the model. Statistical analyses were performed using IBM SPSS Statistics version 26 (SPSS Inc.), R software version 4.2.1 (R Foundation for Statistical Computing; www.R-project.org), and GraphPad Prism 8 (GraphPad Software, Inc.). A two‐sided significance level of 0.05 was used.

## 3 RESULTS

### 3.1 Patient characteristics

The clinical data of 507 patients with LCG who received GI + PPI treatment included age, gender, lesion size, laryngopharyngeal reflux, chronic cough, history of previous surgical resection, history of intubation, and basic patient data (Table 1). Starting from the first treatment, the CC rate was 3% (14/507) in 1 month, 13% (65/507) in 2 months, 33% (165/507) in 3 months, 52% (266/507) in 4 months, 73.6% (373/507) in 5 months, and 86.8% (440/50) in 6 months.

**TABLE 1:** Baseline characteristics

	GI + PPI, *n* = 507 (100%)
Age (year), mean ± SD	45.5 ± 8.59
Gender	
Male	445 (87.8%)
Female	62 (12.2%)
Laryngopharyngeal reflux	
Yes	325 (64.1%)
No	182 (35.9%)
Chronic cough	
Yes	225 (44.4%)
No	282 (55.6%)
Size	
Larger size	205 (40.4%)
Smaller size	302 (59.6%)
History of intubation	
Yes	69 (13.6%)
No	438 (86.4%)
History of surgical resection	
Yes	203 (60.0%)
No	304 (40.0%)

*Note*: GI + PPI, local glucocorticoid injection combined with an oral proton‐pump inhibitor.

### 3.2 Score development

**FIGURE 2:**
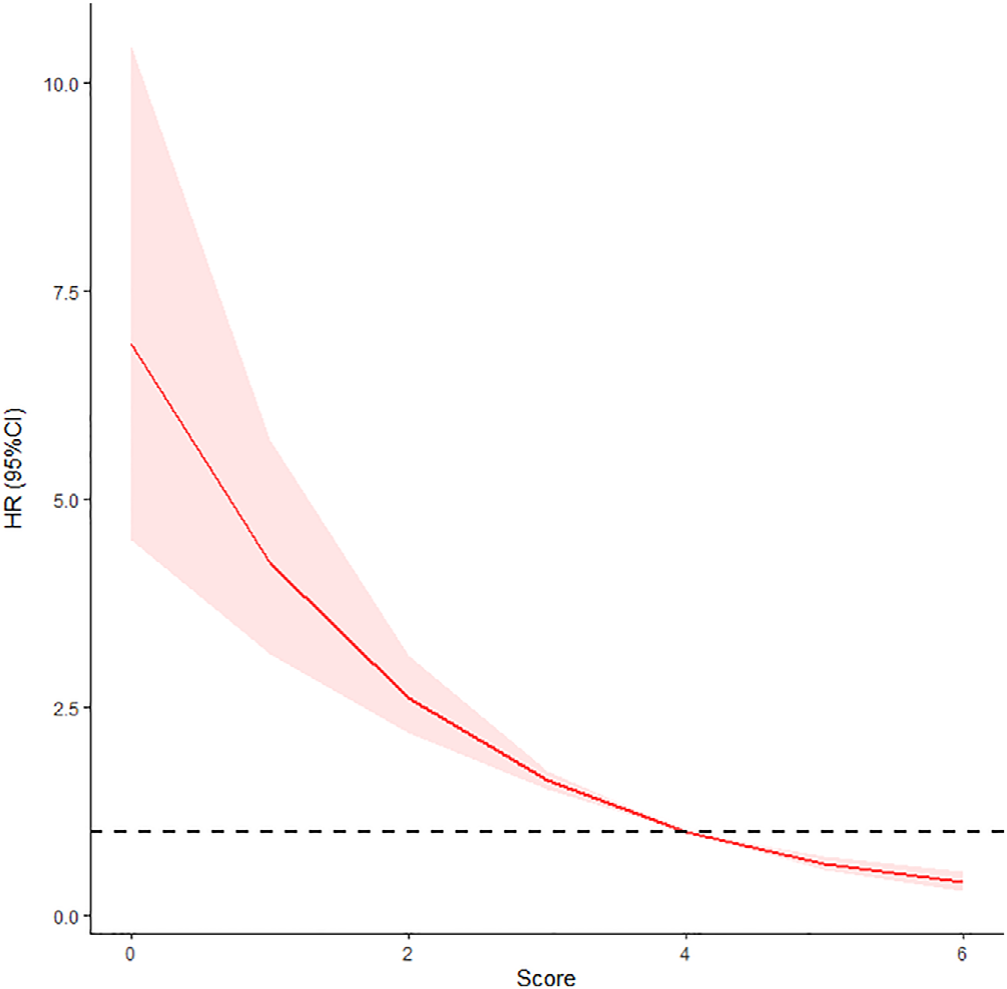
Restricted cubic spline analysis used to determine the cutoff for the RCGSG score

**FIGURE 3:**
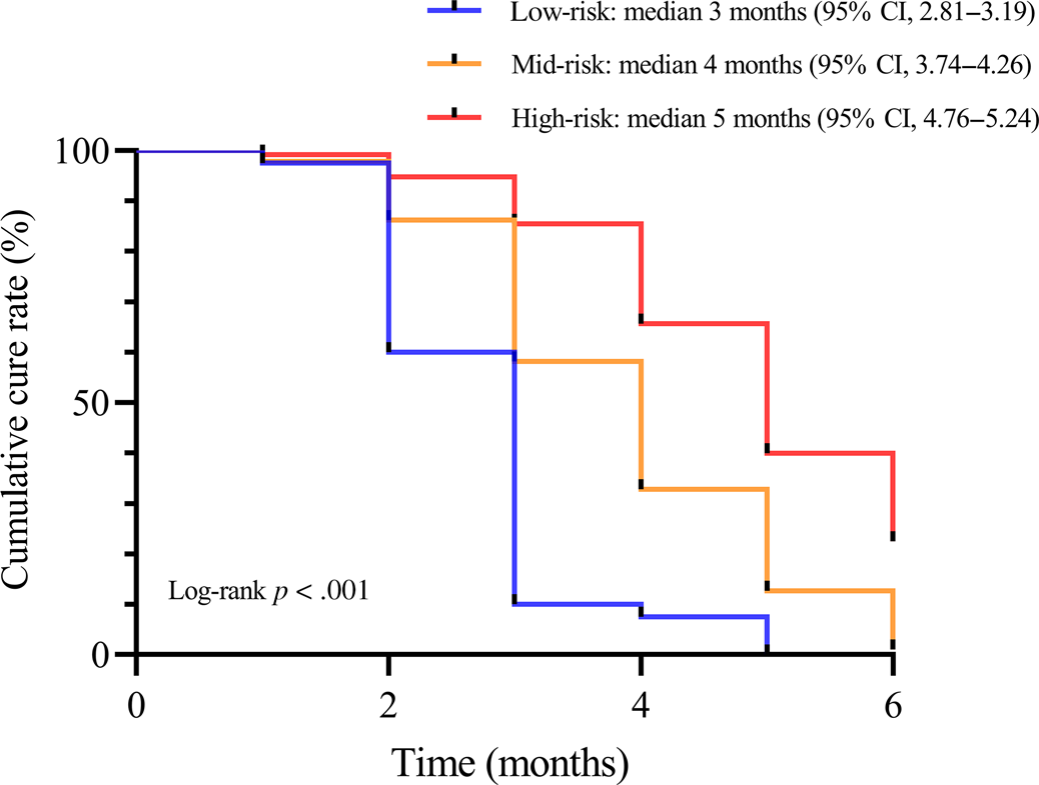
Kaplan–Meier survival curve according to different groups

After univariate and multivariate analyses, male gender [hazard ratio (HR) 0.546, *p* < .001], laryngopharyngeal reflux (HR 0.702, *p* = .001), chronic cough (HR 0.709, *p* = .001), and history of surgical resection (HR 0.433, *p* < .001) were found to be the independent risk factors affecting the prognosis of LCG (Table 2). The independent risk factors screened by the multifactorial analysis were assigned a score based on the regression coefficients. Positive laryngopharyngeal reflux was assigned a score of 1, positive chronic cough was assigned a score of 1, male gender was assigned a score of 2, and history of surgical resection was assigned a score of 2. Therefore, the score was named the RCGSG score (**R**eflus, **C**ough, **G**ender, and **S**urgery in **G**I + PPI Therapy). Restricted cubic splines were drawn according to the score, and the cutoff value was 4 points (Figure 2). The score of 4–6, 2–3, and 0–1 indicated the high‐risk group, moderate‐risk group, and low‐risk group, respectively (Table 3). The survival curves of different risk groups were drawn by the Kaplan–Meier method. The median CC was 3 months (95% CI 2.81–3.19) in the low‐risk group, 4 months (95% CI 3.74–4.26) in the moderate‐risk group, and 5 months (95% CI 4.76–5.24) in the high‐risk group (*p* < .001 using the log‐rank test; Figure 3).

**TABLE 2:** Univariate and multivariate Cox regression analyses

	Univariate		Multivariate		
	HR	*p*	B	HR	*p*
Laryngopharyngeal reflux	0.618	<.001	−0.354	0.702	.001
Chronic cough	0.678	<.001	−0.344	0.709	.001
Gender	0.431	<.001	−0.605	0.546	<.001
History of previous surgical resection	0.407	<.001	−0.836	0.433	<.001
Size	1.094	.368			
History of intubation	1.796	<.001	0.126	1.135	.442

**TABLE 3:** Risk group

Group	Score	Number of cases (%)
Low‐risk group	0–1	42 (82.8%)
Moderate‐risk group	2–3	199 (39.4%)
High‐risk group	4–6	266 (52.5%)

### 3.3 Score validation

**FIGURE 4:**
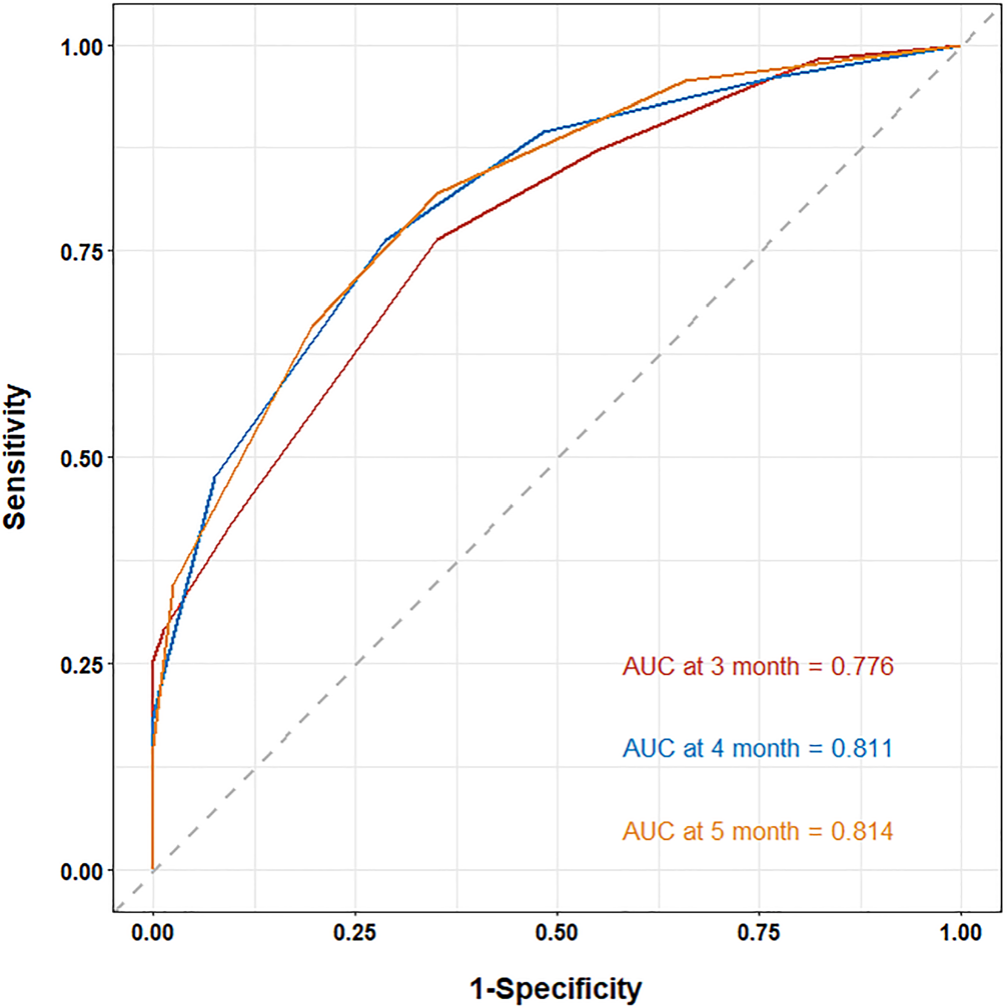
ROC values after 3, 4, and 5 months used to measure the discrimination of RCGSG scores, with a value of >0.75 indicating good discrimination. ROC, receiver‐operating characteristic

**FIGURE 5:**
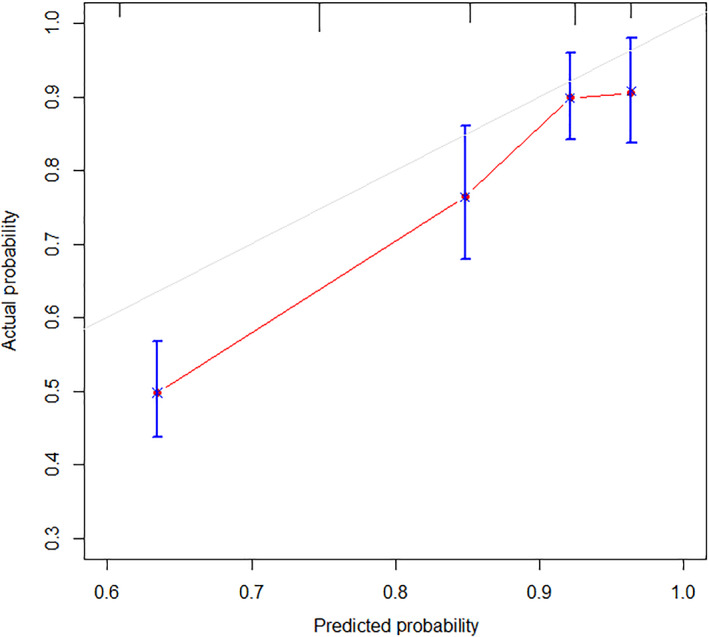
*X*‐axis represents the probability of the outcome event predicted using the model, and the *y*‐axis represents the probability of the outcome event actually observed. A gray line with slope 1 is drawn as the ideal curve for reference. The score model curve drawn using the bootstrap method is marked in red (*b* = 200 replicates)

**FIGURE 6:**
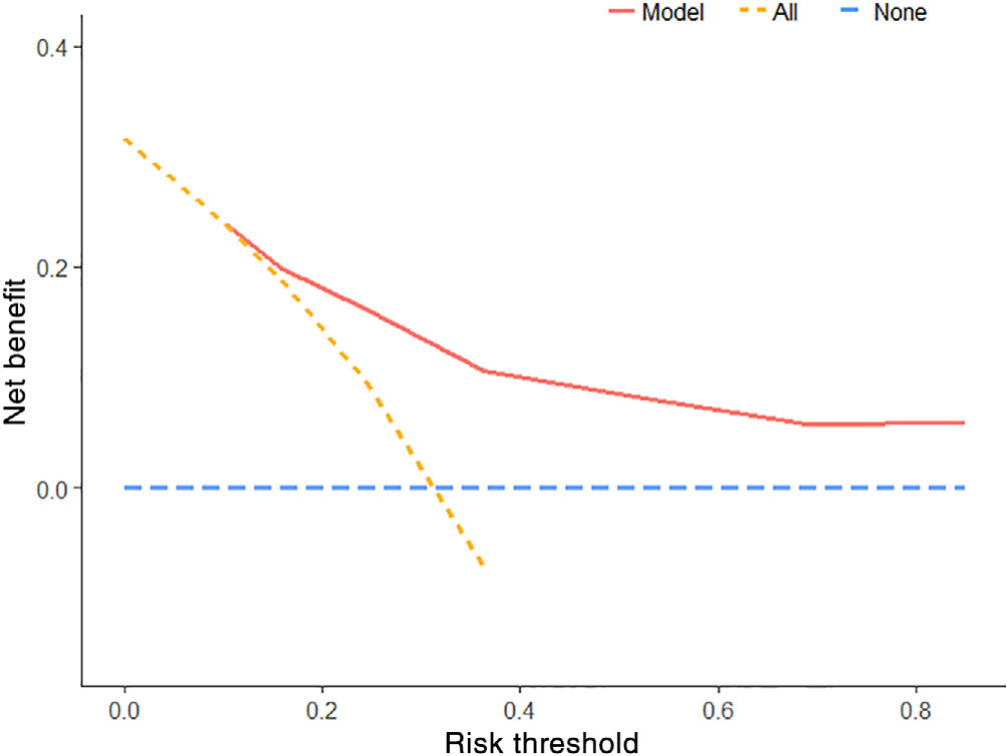
Decision analysis curves for evaluating clinical applicability, with risk thresholds on the *x*‐axis and net benefits on the *y*‐axis. The red curve represents the net benefit when the intervention is applied according to the score model, the orange curve represents the net benefit when all the interventions are applied, and the blue curve represents the net benefit when none of the interventions is applied

The AUC of the RCGSG score was 0.776, 0.811, and 0.814 after 3, 4, and 5 months, respectively, and the AUC >0.75 indicated a good discrimination ability (Figure 4). The calibration curve showed the concurrence of the predicted probability with the actual probability (Figure 5). Finally, we plotted the decision curve to evaluate the clinical applicability of the RCGSG score. The red curve represented the net benefit of intervention and the model, the orange curve represented the net benefit of all interventions, and the blue curve represented the net benefit of no intervention at all. It was seen that the clinical intervention based on the RCGSG score could obtain higher net benefits (Figure 6).

## 4 DISCUSSION

At present, the difficulties in treating LCG are well recognized. Also, each treatment regimen is associated with difficulties.^2^, ^8^, ^9^, ^10^, ^11^ A review of published studies on common treatment regimens revealed that all treatment regimens for LCG required a long treatment period. Arbitrary changes in treatment regimens during the treatment period are not accepted in treating any disease. Botulinum toxin A injection seems to be a good regimen, but hoarseness, choking while drinking water, and other side effects may develop 1 month or even longer after the injection.^12^ At present, a treatment regimen for patients with LCG is chosen randomly in a blinded manner. Even if the first treatment regimen is not found to be effective, it is still continued for more than 6 months before switching to another regimen, which involves a lot of time and money and causes physical and psychological damage to patients. Wang et al. first reported a 60% cure rate 6 months after the GI regimen, whereas Tian et al. further reported a 91% cure rate 6 months after the GI + PPI regimen.^4^, ^5^ However, still some patients were not cured even after a treatment of up to 25 months. Therefore, we decided to develop the RCGSG score to screen out patients for whom GI + PPI treatment was difficult before they chose another treatment option.

We initially included factors such as laryngopharyngeal reflux, chronic cough, history of previous surgical treatment, history of intubation, gender, age, and size. After multifactorial analysis by stepwise regression, only laryngopharyngeal reflux, chronic cough, history of previous surgical treatment, and sex were found to be the independent risk factors for prognosis. Therefore, we also included only these four independent risk factors in the final development of the score. This was consistent with the findings of previous studies.

At present, few prediction models are used to predict the prognosis of LCG. This may be because the influencing factors of treatment are complex. Laryngopharyngeal reflux is one of the possible risk factors. The arytenoid cartilage region lacks the acid‐fast barrier in the stomach and is vulnerable to the effect of gastric acid, pepsin, and other substances. The 24‐h combined dual‐channel impedance/pH‐metry (24‐h MII‐pH) is considered by many scholars as the gold standard for the diagnosis of laryngopharyngeal reflux.^13^ Ylitalo et al. used 24‐h MII‐pH in patients with LCG; up to 65% of patients were positive.^14^ Koufman et al. suggested that the persistence of laryngopharyngeal reflux might lead to the organization of LCG and increase the difficulty in treating the disease.^15^

Mechanical factors are another type of risk factors for the prognosis of LCG. Chevalier Jackson et al. first suggested in 1935 that the development and persistence of LCG might be related to the impingement of the arytenoid cartilage, and compared this phenomenon to “a hammer striking an anvil.”^1^ Damrose et al., in 2008, further speculated that the possible causes for the occurrence and persistence of LCG were the initial trauma resulting in incomplete arytenoid cartilage membrane and the subsequent “traumatic exposure” caused by persistent chronic coughing, throat clearing, and voice abuse, making it difficult to restore the integrity of the cartilage membrane.^16^ Chronic cough was the most easily observed symptom by patients compared with several other symptoms. Therefore, only one mechanical factor, chronic cough, was included in our study.

In the past, the effect of surgery on the prognosis of LCG received little attention. Researchers often regarded surgery as the treatment option for LCG, but they ignored the effect of surgery as a trauma on the prognosis of LCG. Surgery, as a treatment option for LCG, creates a larger mucosal defect. Our previous studies demonstrated that a history of surgical resection was an important factor in the prognosis of LCG.^10^, ^17^

Gender is another factor associated with the prognosis of LCG. Many scholars reported that female patients always had better treatment outcomes.^18^ In 2020, Zhang et al. reported an overall 93% effective rate 3 months after GI treatment in 14 female patients.^19^ This might be because female glottic anatomy was more prone to post‐intubation injury, with no other persistent risk factors, resulting in a more treatable post‐intubation granuloma.

The lack of external validation should be acknowledged as a gap because this was a single‐center study. We expected other research centers to validate this predictive score. However, the RCGSG score we developed is still the most simple and practical score to predict the prognosis of LCG. The RCGSG score can be used to determine the prognosis of patients with LCG treated with GI + PPI in 1 min. For example, the use of the GI + PPI regimen may be inappropriate for patients in the high‐risk group. Patients in the high‐risk group should be switched to other treatment options, or at least should no longer be treated with invasive GI regimens. We recommend the GI + PPI regimen for patients in the low‐risk group because the median CC is only 3 months.

## 5 CONCLUSION

The RCGSG score, developed based on laryngopharyngeal reflux, chronic cough, gender, and surgical resection history, has been internally verified to be a good predictor of the prognosis of patients with LCG receiving GI + PPI treatment.

## FUNDING INFORMATION

None.

## CONFLICT OF INTEREST

None reported.
